# Artificial Intelligence in Teledentistry

**DOI:** 10.15190/d.2022.12

**Published:** 2022-09-30

**Authors:** Panchali Batra, Himanshu Tagra, Sakshi Katyal

**Affiliations:** ^1^Department of Orthodontics, Faculty of Dentistry, Jamia Millia Islamia, New Delhi, India; ^2^HCL Technologies Ltd, New Delhi, India

**Keywords:** Artificial Intelligence (AI), teledentistry, remote dentistry, facial expression analysis, machine learning, deep learning, social media analysis.

## Abstract

Artificial intelligence (AI) has grown tremendously in the past decade. The application of AI in teledentistry can reform the way dental care, dental education, research, and subsequent innovations can happen remotely. Machine learning including deep learning-based algorithms can be developed to create predictive models of risk assessment for oral health related conditions, consequent complications, and patient stratification. Patients can be empowered to self-diagnose and apply preventive measures or self-manage some early stages of dental diseases. Applications of AI in teledentistry can be beneficial for both, the dental surgeon and the patient. AI enables better remote screening, diagnosis, record keeping, triaging, and monitoring of dental patients based on smart devices. This will take away rudimentary cases requiring run-of-the-mill treatments from dentists and enable them to concentrate on highly complex cases. This would also enable the dentists to serve a larger and deprived population in inaccessible areas. Its usage in teledentistry can bring a paradigm shift from curative to preventive personalised approach in dentistry. A strong asset to teledentistry could be a robust and comprehensive feedback mechanism routed through various channels proposed in this paper. This paper discusses the application of AI in teledentistry and proposes a feedback mechanism to enhance performance in teledentistry.

## SUMMARY

*1. Introduction*

*2. Advantages of Teledentistry*

*3. Limitations of Teledentistry*

*4. Platforms for Teledentistry*

*5.* *Applications of artificial intelligence in Teledentistry*

- *5.1 Screening, Diagnostic Workup and Triaging*

- *5.2 Clinical Decision Support and Follow-Up*

- *5.3 Feedback Mechanism*

*6. Conclusion*

## 1. Introduction

Oral healthcare is one of the most neglected areas of healthcare. Dental caries followed by periodontal diseases are the most prevalent dental pathologies and are the major reasons to visit a dental clinic^1^.

According to the World Health Organization (WHO), dental caries affect 60-90% of schoolchildren and most adults, i.e., 2.40 billion adults and 621 million children are affected by dental caries^2^. Despite its high prevalence, there is a vast percentage of the population which do not have direct access to dental clinics. Oral problems add directly or indirectly to the economic burden of the world. Indirectly, dental problems lead to absenteeism from work. Worldwide, oral diseases lead to a treatment cost of US$298 billion yearly and an indirect loss of US$144 billion yearly^3^.

**Figure 1:**
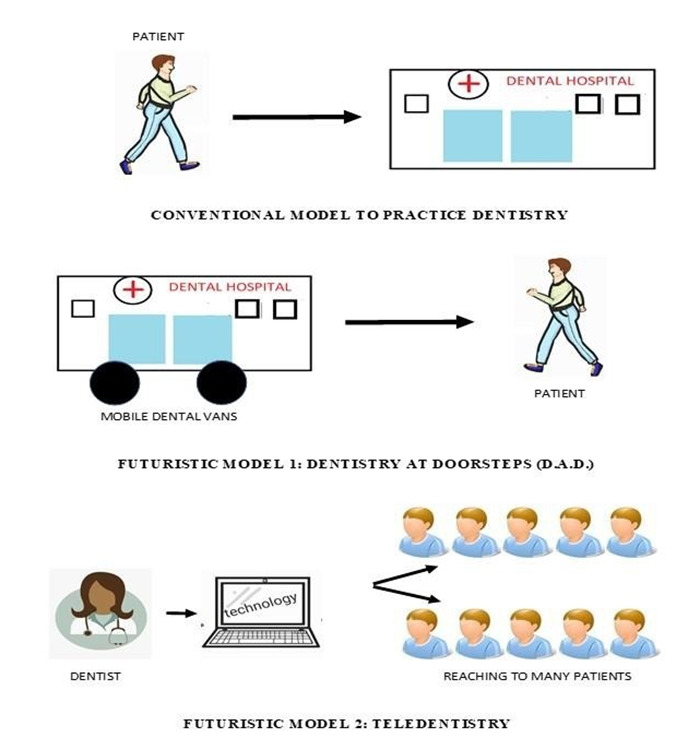
Conventional and futuristic models of dentistry

Dental care traditionally has been rendered through a hospital-based model in which the patient reports to a dental clinic/hospital when he experiences any dental or oral health-related problem. In the coming years, this conventional model will reform in two big ways either with dentistry being delivered at doorstep (DAD-Dentistry at Doorsteps) through mobile dental vans or dental care being provided remotely and proactively through Information Technology (IT), and telecommunication-based platforms (Figure 1). The term “Teledentistry” is derived from two words “tele” which means distance and “dentistry” which is the branch of medicine concerned with diagnosis and treatment of problems of teeth and surrounding tissues. Thus, teledentistry can be defined as “practising dental care and education remotely i.e., diagnosing and rendering care, or teaching dentistry from a distance via any telecommunication platforms such as phone, computers, e-mail, radio broadcasting, television, etc.” This term was first defined by Cook in 1997 as “the practice of using video-conferencing technologies to diagnose and provide advice about treatment over a distance^4^”. Teledentistry in the coming years will be smarter through the incorporation of AI. Artificial intelligence in Dentistry can be defined as “the science that would create smart machines and systems that would simulate human intelligence based on pre-fed algorithms, and would be able to assist dental clinicians, academicians, and researchers in their respective activities^5^”. Machine learning is a subset of AI, in which the systems can automatically learn from experience rather than from the data fed/programmed in them. Further advancement in AI led to the development of Deep learning, in which the system uses multiple processing layers of the data, just as the neural networks of the brain, and based on the difference in perception of layers, interpretation is provided^6,7^. For example, a computer will be able to suggest by reading a radiograph, based on the difference in radiopacities, that a particular tooth is carious. AI-based systems can assist a clinician in screening, diagnosing and decisions making. AI has been used in medicine in surveying, monitoring, data management, medical education, health promotion, consultation, triaging, imaging, treatment, self-management, drug development, surgery, predicting hospital mortality rate, global health risks, forecasting pandemics, measuring treatment effectiveness, medical statistics, and cybersecurity of these telemedicine platforms^8^. AI has been proven useful for the diagnosis and management of many acute and chronic conditions such as diabetes, dermatological conditions, cardiac conditions, stroke care, ophthalmological conditions etc^9-12^.

## 2. Advantages of Teledentistry

The concept of teledentistry has many advantages such as it can be beneficial to areas where there is a dearth of healthcare professionals, so a patient can connect with a doctor far away nationally and even internationally. Patients can take a second opinion and can connect with any specialty easily. It also saves resources, money and energy spent on travel and consultation, hassles of long queues at tertiary care centres^5^, saves on time and absentia from work or school, and has an economic impact on the nation. In case of emergency, or odd hours, or during pandemics as COVID-19, such services are highly beneficial. It has the potential to shift the ecosystem from curative to preventive treatment, through advancements in technology^6,7^. Follow-up of high-risk or differentially abled groups and service to the underserved will be more efficient through these platforms.

## 3. Limitations of Teledentistry

Though Teledentistry has helped in providing dental services during the pandemic, it comes with inherent disadvantage of altering the relationship between patients and health professionals thus creating challenges to its acceptance in full potential. The limited effectiveness of teledentistry can be attributed to the following reasons: lack of digital literacy amongst some sections of population, insufficient infrastructure and internet services, lack of universal access, concerns about data privacy and data security and lack of stringent guidelines to regulate the digital health system. Teledentistry is yet to explore its full potential because of these limitations.

## 4. Platforms for Teledentistry

Teledentistry can be practised through many dedicated healthcare platforms. These platforms are compliant with the Health Insurance Portability and Accountability Act (HIPAA) guidelines and ensure that the patient’s information is end-to-end encrypted and secure. Before using common platforms such as skype, zoom, google meet, etc., one needs to configure the settings to make it HIPAA compliant and practice through dedicated versions as “zoom for healthcare”, “G Suite” by Google meet. Some dedicated platforms for teledentistry which employ artificial intelligence, such as Dentulu^13^, Toothpic^14^, Rhinogram^15^, Review tools^16^, Smile snap^17^, Teledentix^18^, TeleDent^19^, Carestack^20^, Dental Monitoring^21^, are available that can assist a clinician in various steps as diagnosis, sharing virtual records, providing alerts for potential complications, virtual monitoring of the progress of the case, make payments, and promote dental health awareness. One must be careful while using WhatsApp, Apple FaceTime, and Facebook Messenger as these are not HIPAA compliant^22^.

## **5.** Applications of artificial intelligence in Teledentistry

**Figure 2:**
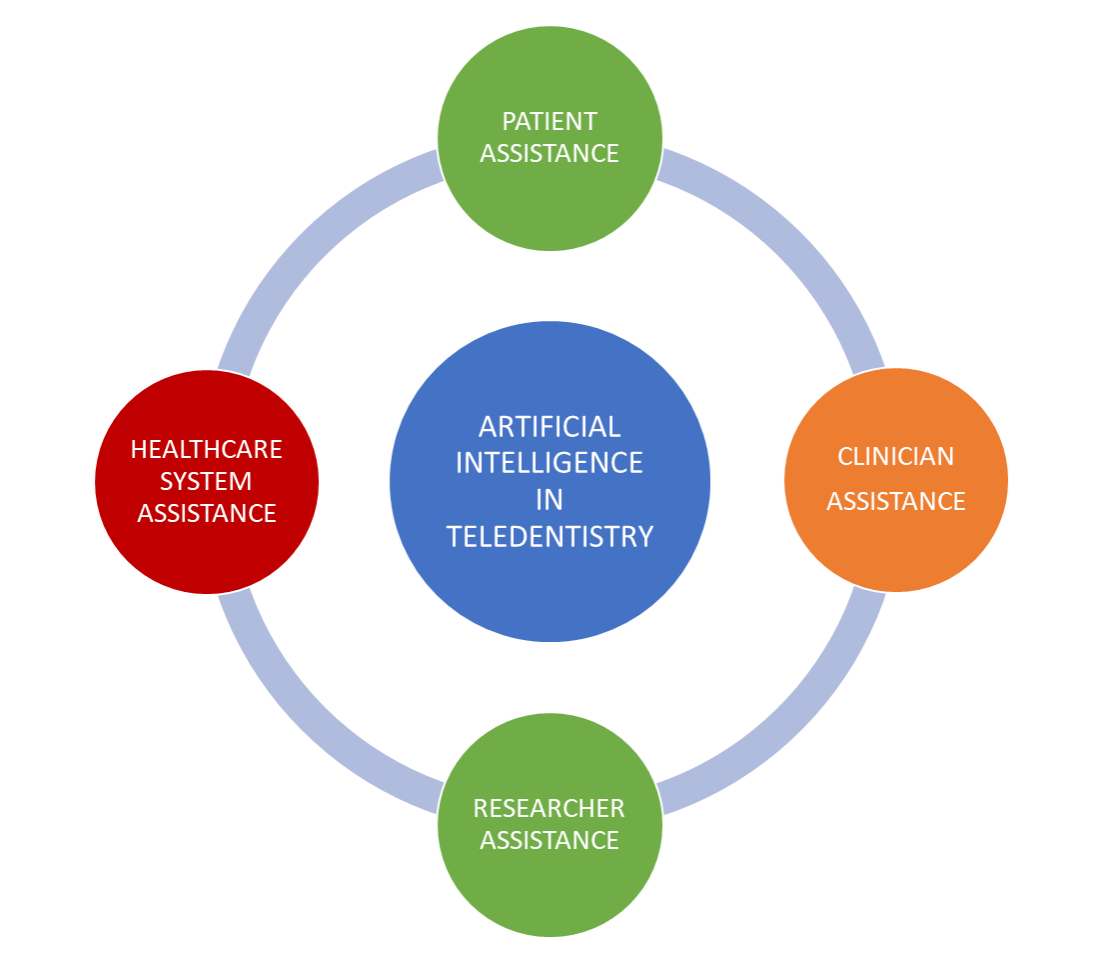
Application of Artificial Intelligence in Teledentistry

Artificial intelligence can assist patients, clinicians, researchers, and health care systems (Figure 2). The main applications can be summed up as follow.

### 5.1 Screening, Diagnostic Workup and Triaging

Remote oral screening can be highly beneficial in shifting the approach from curative to preventive treatment. Regular screening of the population could lead to a tremendous decline in oral health-related problems. Most common oral diseases such as caries can be prevented at the first sign of demineralization if diagnosed timely. Remote oral surveillance can also be beneficial in screening for deadlier diseases such as oral cancers which would help in reducing the mortality and morbidity associated with malignant and premalignant lesions by early diagnosis. Oral cancers can be prevented by identifying high-risk populations and regular remote oral screenings. AI has been used to predict patients who are at high risk of oral cancers based on their risk factors, habits, and sociodemographic and genomic data^23^. Remote screening with simple methods such as intraoral photographs have been useful in identifying Oral squamous cell carcinoma, leukoplakia, and lichen planus lesions^24^. Apart from intraoral photograph-based remote diagnosis, a smartphone based auto-fluorescence oral cancer probe has been devised to screen for oral cancers and triage them according to needs using AI^25^. These probes can be installed at primary centres in high-risk populations and will enable remote consultations. These probes can be operated even in the absence of any trained healthcare worker. This would also enable predictive population risk stratification to reduce the burden of oral diseases.

AI has also immensely contributed to remote forensic dentistry. The software can predict the age of the patient^26^ based on the medical images or radiographs and full-face reconstruction can be carried out through lateral cephalograms with the help of machine learning models^27^.

Radiographs are an important diagnostic tool for a dentist and can be easily shared remotely for teleconsultation. With the advent of digital radiographs, it is possible to measure the length, angles, position, and density of objects. A digital radiograph consists of many pixels and each pixel emits a particular brightness^28^. Intelligent apps and software programs can read and interpret radiographs based on the difference in radiopacities. The application can suggest the presence of dental caries^29^, changes in bone level in periodontal diseases and are useful in cephalometric analysis during orthodontic diagnostic workup which is then verified by clinician remotely. Since the depth of the lesion can also be calculated through AI-based programs, the software can even suggest a few treatments plans as when and how to restore the carious tooth. Similar to 2D radiographs, 3D CBCTs have the inbuilt algorithms to mark bones as maxilla, and mandible, generate cephalograms from CBCT, and interpret them, thus saving the time and efforts of a clinician. The digital scans, which are now readily available, use artificial intelligence for the diagnosis of various dental conditions such as filled teeth, gingivitis, periodontitis, crowned teeth, and implant^30^.

Apart from clinical diagnosis, AI can add to tissue diagnosis by capturing high-resolution images of the biopsy slides which can then be reviewed by a remote pathologist^31^.

### 5.2 Clinical Decision Support and Follow-Up

Moving over from diagnosis, AI has contributed immensely to remote decision-making. Many softwares have been developed to assist clinicians in decision-making based on deep learning algorithms. Some simple decision-making softwares are available for fillings, to mark the margins of a tooth preparation scan for fabricating prosthesis, smile designing, suggesting extraction or non-extraction treatment in orthodontics, to assess the treatment outcomes, orthodontic or surgical treatment simulation, to monitor the progress of the orthodontic treatment, to identify bone loss through X rays and type of periodontitis by examining subgingival plaque. Apart from the tele-communication platforms mentioned above some dedicated AI-assisted monitoring or follow-up software are available. One such software is Dental monitoring. This software can be used to remotely monitor the amount of tooth movement, breakages, wire engagement, etc. Software to remind the patient for medication, next appointment, and payments has also been developed^32^.

### 5.3 Feedback Mechanism

Patient feedback is considered an integral initiative for facilitating reflective practice which could lead to overall quality improvement and professional development. Typically, the feedback is collected as part of a questionnaire-based assessment. Patient Feedback is now increasingly becoming part of medical education as well as medical revalidation. In a typical consultation, feedback mechanisms are twofold namely through face-2-face interaction as well as formal questionnaire based. The advent of teledentistry has given a totally new paradigm to doctor and patient behavior. Remote interactions do not come naturally both to the doctor as well as the patient. Medical education also does not cover the aspect of remote interactions. To bring about continuous improvement in the interaction of a doctor in teledentistry, there is a need for a continuous quick turnabout-based feedback mechanism. Standard questionnaire-based assessment with turnabout after fixed long intervals will not suffice.

We propose an AI-based framework to gather feedback and enable the improvement of doctor consultation in teledentistry. The feedback can be categorized into 4 types:

· Form-based feedback

· Facial expression-based feedback

· Patient adhesion-based feedback based

· Social media-based feedback

#### a. Form-Based Feedback

An AI (implemented using Case-Based Reasoning) based mobile application which can intelligently ask questions to the patient based on the profile of the patient, chronology of visits, answer to the previous questionnaire, etc can be developed to know the level of satisfaction of the teleconsultation. The applications analyze the answers and based on that rates the interaction.

#### b. Facial expression-based feedback

Patient emotion detection is very important during remote interaction. Emotion detection can be done based on verbal as well as non-verbal communication mechanisms. Typically, in remote learning, healthcare, etc., non-verbal communication mechanisms are more suitable for emotion detection. The facial expression of the patient gives the type of engagement experienced by him. Typical expressions experienced by a patient can range from fear, happiness, sadness, anger, or confusion^33^. Deep learning networks (DLN) with convolutional neural networks (CNN) are used for detecting and analyzing facial expressions^34^. These models are already being used in remote teachings^35^. The facial expression evaluation can be executed on patients as well as doctors. It gives the expression and engagement level of the doctor while interacting with the patient. For example, was the doctor angry or pleasant during the interaction, or did the doctor pick up any mobile calls could be the possible scoring factors for doctors.

#### c. Patient adhesion-based feedback

AI-based software can be developed to analyze the dropouts of scheduled appointments as they could also point toward the attrition of patients. AI could also be used to assess the potential timing of the next appointment for each patient. This will be based on many factors such as oral health of the patient, oral habits, potential disease occurrence based on the current state of the teeth, the overall health of the patient (other comorbidities), etc. Based on the above parameters, if the patient misses the appointment, it would be considered a lost patient. Statistical analysis of the result of a group of patients would assess the quality of teledentistry.

#### d. Social media-based feedback

The advent of various social forums has given rise to patients discussing freely the quality of interaction with the clinic, hospital, and doctor and rating them. Twitter is currently the most popular form of social media used for healthcare communication^36^. Various intelligent web crawlers scrap these sites for information and give feedback. Web Scraping is a process to extract data from the internet using any method or technique^37^. These forums are also useful in building the confidence of a patient if he/she is going for a complicated treatment.

## 6. Conclusion

Developments in AI-assisted teledentistry are rising enormously and point towards a digital era that would make all dentistry-related avenues intelligent.

AI-assisted teledentistry has the potential to perform better than the eyes of a trained or overworked clinician. The current applications of teledentistry are mainly adjunctive rather than a replacement for face-to-face consultation. Artificial intelligence has the potential to shift the trend from a curative to a predictive and preventive personalized model. From this quantum change which will continue to evolve, going further, AI will assist clinicians, academicians, and researchers in all spheres of dentistry. This change could be enabled by introducing digital literacy in the dental education curriculum and workforce.
